# Practical Notes on Diagnosis and Treatment

**Published:** 1910-08-06

**Authors:** 


### Practical Notes on Diagnosis and Treatment.

**Iron Preparations.**

I am convinced that in chlorosis the ordinary inorganic preparations of iron cure much more quickly than organically combined iron does; patients fed on even a rich and varied diet containing plenty of organic iron do not as a rule recover until inorganic iron is administered.—*Professor Stockman.*

**Pleural Effusion.**

When the fluid in a case of serous effusion has been withdrawn, the application of an ice-bag, continued for some days, always tends to hinder the fresh secretion of fluid. It is an advantage to encourage deep breathing on the part of the patient in order to promote expansion of the lung again.—*Dr. Lees.*

**The Gouty Inheritance.**

However difficult it may be to understand, observations make it certain that the offspring of gouty parents are specially liable to the painful neuroses, and this is particularly true of those who are of the female sex. Thus in the more marked forms of migraine, parental or ancestral gout is seldom absent.—*Sir Wm. Gowers.*

**Warts.**

The best internal medicine for getting rid of warts is arsenic. Small doses of magnesium sulphate are often successful. As a local application one part of corrosive sublimate to 25 parts of flexile collodion is suggested.—*Dr. Graham Little.*

**Injury to the Back.**

Never treat lightly a case of injury to the back, no matter how trivial the cause may be. If there be the least indication of interruption of bladder power, or if there be ever so little tingling about the hands, feet, or trunk, such a case may prove disastrous and should be treated at once as being serious.—*Sir Wm. Bennett.*

**Nitrate of Silver.**

This drug is still used in small doses as a sedative to the stomach: for this purpose about $\frac{1}{10}$ grain is the usual dose. After absorption nitrate of silver has a tonic action on the nervous system. As an antidote, or as a means of relieving the unpleasant taste left after using the solid form locally on the throat, a solution of common salt is effective.—*Sir T. Lauder Brunton.*

